# Organic and inorganic mixed phase modification of a silver surface for functionalization with biomolecules and stabilization of electromotive force†

**DOI:** 10.1039/d1ra03449a

**Published:** 2021-07-16

**Authors:** Miyuki Tabata, Chiho Kataoka-Hamai, Kozue Nogami, Daiju Tsuya, Tatsuro Goda, Akira Matsumoto, Yuji Miyahara

**Affiliations:** Tokyo Medical and Dental University 2-3-10 Kanda-Surugadai, Chiyoda Tokyo 101-0062 Japan; National Institute for Materials Science 1-2-1 Sengen, Tsukuba Ibaraki 305-0047 Japan

## Abstract

A solid-state potentiometric biosensor based on the organic and inorganic mixed phase modification of a silver surface is proposed. Stabilization of the electromotive force and functionalization with biomolecules on the sensing surface were simultaneously achieved using silver chloride chemically deposited with 1,3-diaminopropanetetraacetic acid ferric ammonium salt monohydrate and a self-assembled monolayer with oligonucleotide probes, respectively. The formation of silver chloride and adsorption of alkanethiol on the silver surface were confirmed with X-ray photoelectron spectroscopy. The resulting modified surface reduced the nonspecific binding of interfering biomolecules and achieved a high signal to noise ratio. The electromotive forces of the modified silver thin film electrodes were stable under constant chloride ion concentrations. Hybridization assays were performed to detect microRNA 146. The lower limit of detection was 0.1 pM because of the small standard deviation. The proposed biosensor could be useful as a disposable single-use sensor in medical fields such as liquid biopsies.

## Abstract 2

The organic and inorganic mixed phase modification of a silver surface is proposed for solid-state potentiometric biosensors.

## Graphical abstract

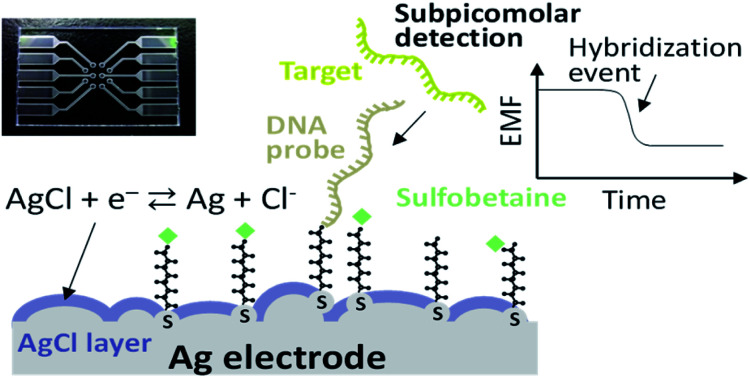

## Introduction

There has been increasing interest in solid-state electrochemical biosensors for detecting biomolecular recognition. Various types of electrochemical sensors have been developed to realize simple, rapid, sensitive, inexpensive, and multiplexed analyses of biomarkers for point-of-care-testing.^1,2^ Simple electrode systems consisting of a working electrode, counter electrode, and reference electrode are capable of signal transduction from a molecular recognition signal to an electrical signal, sometimes in combination with nanoparticles, nanotubes, or graphene.^3,4^ This method is therefore suitable for the miniaturization of detection systems, mass-production of sensors, and integration of sensors for parallel multi-analyte detection. The electrochemical or electrical signals are generated directly *via* the properties of the products of biomolecular recognition or with the use of electroactive reporter molecules. Gold, platinum, graphite, and glassy carbon are the most frequently used materials for the working electrode and counter electrode because they are chemically inert and stable against redox reactions at the surface. Silver/silver chloride is usually used for a reference electrode.

To functionalize the metal surface of the working electrode for biosensing, the strong affinity between sulphur and transition metal surfaces is usually used. Organosulfur compounds such as alkanethiols and alkyl disulphides coordinate strongly to gold,^5–7^ silver,^5,8^ copper,^9^ and platinum.^10^ Biomolecules or probe molecules are then immobilized using functional groups at the terminal of the self-assembled monolayer (SAM) for molecular recognition on the working electrode. Electrochemical biosensors based on voltammetric, impedimetric, amperometric, and coulometric measurements can be achieved using these electrode materials and configuration.^11–13^

Among electrochemical sensors, potentiometric sensors are suitable for miniaturization, and therefore high-density integration, because their signals do not depend on the surface area of the electrode. pH sensors are a typical example of a potentiometric sensor and are frequently used as transducers in biosensors. An array of ion-sensitive field effect transistors was applied to the detection unit in a reported DNA sequencer.^14^ Transition metals are not usually used as materials for potentiometric measurements. This is because the surfaces of transition metals such as gold, platinum, or silver are polarized and the electromotive force (EMF) at the metal/aqueous solution interface is not electrochemically defined. Drifts in the EMFs of transition metal electrodes are usually observed in potentiometric measurements because of the lack of electrochemical equilibrium. Even if a SAM is formed on the surface of transition metal electrodes, the quality of the SAM is unlikely to be ideal. This is because transition metal electrodes used for electrochemical measurements are polycrystalline and have a grain and surface roughness. This situation differs from that when using single crystal substrates such as Au (111) or Ag (111).^15,16^ Defects and disorders are therefore formed in the SAM,^17,18^ which would influence the stability of the EMF of the SAM/metal electrode, reflecting the potential behaviour of the underlying metal.

Instead of transition metals, metal oxides such as Ta_2_O_5_, HfO_2_, SiO_2_, and Si_3_N_4_ are usually used as sensing materials for potentiometric pH sensors. In these materials, silanol groups are formed on the surface in aqueous solution, and dissociation of these silanol groups leads to an electrochemical equilibrium which results in stabilization of the potential at the solid/liquid interface. Although organosilicon derivatives such as alkyl chlorosilanes, alkyl alkoxysilanes, and alkyl aminosilanes are often used to functionalize the metal oxide surface,^5,19,20^ high-quality SAMs of organosilicon derivatives are not simple to produce, partly because of the need to carefully control reaction conditions such as the amount of water in solution^21–23^ and multilayer formation.^24,25^ It is therefore difficult to control the density and orientation of immobilized biomolecules in a reproducible manner.^26^

In the present study, organic and inorganic mixed phase modification of the silver surface is proposed to realize an electrochemically defined interface capable of immobilization of biomolecules with controlled density and orientation. The surface of silver is easily functionalized with biomolecules using a SAM of an alkanethiol. The EMF of the silver surface is easily stabilized by forming silver chloride. The structure and chemistry of alkanethiol SAMs on Ag(111) have been previously investigated in detail.^5,27,28^ The EMF of the silver/silver chloride electrode is stable and constant in the presence of fixed chloride ion concentrations. Here, the SAM/silver and silver/silver chloride structures are mixed and integrated at the surface of a silver thin film to achieve functionalization of biomolecules and stabilization of the EMF of the electrochemical biosensor. This organic and inorganic mixed surface of the silver thin film is applied to the potentiometric detection of nucleic acids for cancer diagnosis in liquid biopsies.

## Results and discussion

### Chemical deposition of silver chloride

Thiols have strong affinity to silver, so the SAM was first formed on the surface of silver, and then silver chloride was chemically formed on the surface of the SAM/Ag substrate. The fabrication process of silver chloride has to be compatible with the SAM/Ag substrate, and a thin layer of silver chloride is required to maintain the functionality of the SAM. The chemical deposition method was used to deposit silver chloride in the present study, to ensure precise control of the thin silver chloride layer, while silver chloride was formed electrochemically in dilute HCl solution by applying a voltage to a platinum counter electrode in the conventional method.

**Fig. 1:**
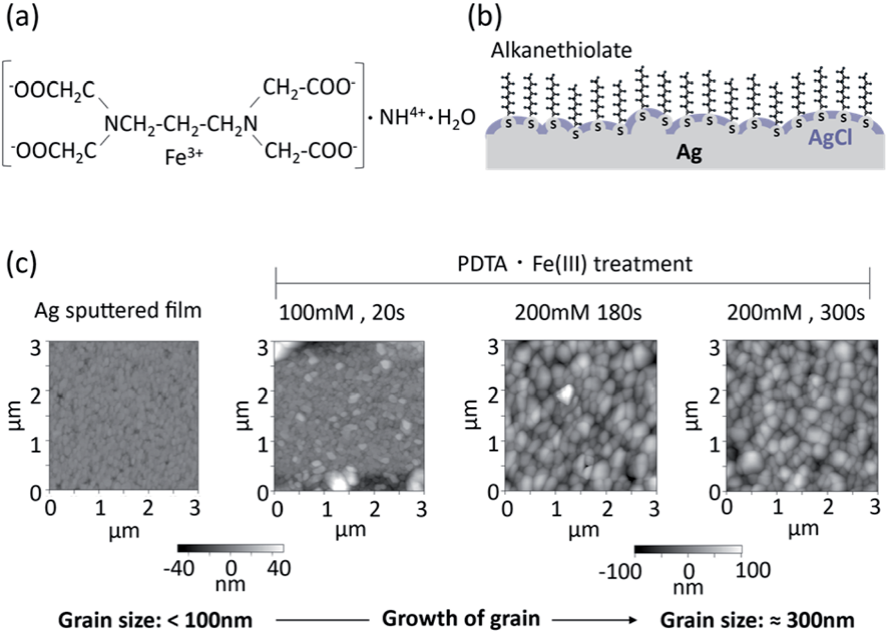
Surface morphology of silver chloride layer prepared by chemical deposition using PDTA·Fe(iii). (a) Chemical formula of PDTA·Fe(iii); (b) conceptual structure of the organic and inorganic mixed phase modification of a silver surface; (c) AFM images of silver chloride surfaces prepared with PDTA·Fe(iii) on a sputtered silver thin film.

Chemical deposition of silver/silver chloride has been reported using 1,3-diaminopropanetetraacetic acid ferric ammonium salt monohydrate (PDTA·Fe(iii)).^29^ The chemical structure of PDTA·Fe(iii) is shown in Fig. 1a. The silver surface is oxidized with PDTA·Fe(iii) to form silver chloride without applying a voltage according to eqn (1):

1 PDTA·Fe^3+^ + Ag + NaCl → PDTA·Fe^2+^ + AgCl + Na^+^

Silver/silver chloride fabricated by chemical deposition with PDTA·Fe(iii) was reportedly applied as an internal electrode in ion sensors.^30^ With this method, it is easy to control the thickness of the silver chloride layer by optimizing the concentration of PDTA·Fe(iii) and reaction time.

A conceptual structure of the organic and inorganic mixed phase modification of the silver surface is shown in Fig. 1b. Alkanethiol groups are in direct contact with the silver surface, and the remaining silver surface is covered with silver chloride. The alkyl chains of the SAM of alkane thiolates on the clean Ag(111) surface are orientated practically perpendicular to the surface, while those prepared on an Au(111) surface are tilted 26–28° from the surface normal.^5^ The SAM of alkane thiolates adsorbed on an evaporated silver surface was also reported to have a much smaller average tilt angle from the surface normal^31^ compared with the Au(111) surface. Functional groups of the alkanethiol can be utilized for the immobilization of biomolecules. The electrochemical equilibrium can be established at the interface between the silver chloride and aqueous solution containing a constant concentration of chloride ions. The equilibrium between the silver metal (Ag) and the silver chloride (AgCl) can be written as follows:

2 Ag^+^ + e^−^ ⇄ Ag

3 AgCl + e^−^ ⇄ Ag + Cl^−^

These equations can be simplified.

4 AgCl ⇄ Ag^+^ + Cl^−^

Based on the above equilibrium, the ion exchange current flows efficiently at the interface between silver chloride and an aqueous solution. The dependence of the electromotive force of the silver/silver chloride electrode on the activity of chloride ions follows the Nernst equation.

Atomic force microscope (AFM) images of sputtered thin films of silver and silver chloride after PDTA·Fe(iii) treatment are shown in Fig. 1c. The grain size of the silver/silver chloride increased with increasing reaction time and increasing PDTA·Fe(iii) concentration. The average grain size of the silver/silver chloride treated with 200 mM PDTA·Fe(iii) for 300 s was approximately 300 nm, while that of the as-deposited Ag film was smaller than 100 nm. The thickness of the silver chloride layer prepared with 200 mM PDTA·Fe(iii) for 180 s was around 280 nm, as shown in Fig. S1, ESI.† To maximize the compatibility with the SAM and accessibility of the target DNA to the oligonucleotide probes immobilized on the silver surface, the silver chloride layer should be as thin as possible, be a pinhole-free and continuous solid layer, and show stable EMF based on electrochemical equilibrium.

### Optimization of silver chloride electrode

**Fig. 2:**
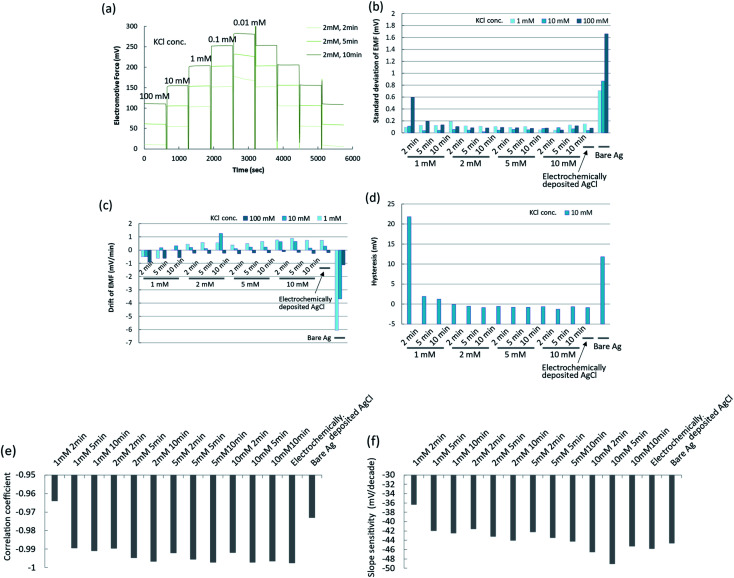
Characterization of silver chloride electrodes prepared by chemical deposition using PDTA·Fe(iii). (a) Time courses of EMFs for silver chloride electrodes prepared under different conditions; (b) standard deviations of EMFs for silver chloride electrodes prepared under different conditions; (c) drifts of EMFs for silver chloride electrodes prepared under different conditions; (d) hystereses of EMFs for silver chloride electrodes prepared under different conditions; (e) correlation coefficients of calibration curves for silver chloride electrodes prepared under different conditions; (f) slope sensitivities of calibration curves for silver chloride electrodes prepared under different conditions.

The conditions of the chemical deposition of silver chloride were optimized using the potentiometric responses as an indicator by changing the PDTA·Fe(iii) concentration and reaction time. The wire-type electrodes prepared under different conditions were evaluated from the slope, fluctuation, drift, and hysteresis of the EMF. Typical raw data for the EMFs of the silver/silver chloride electrodes in response to potassium chloride concentrations of 0.01 mM to 100 mM are shown in Fig. 2a. The response curve of each electrode is shown offset on the voltage axis for clarity. For comparison, the EMF behaviours of a bare silver electrode and an electrochemically deposited silver/silver chloride electrode are shown in Fig. S2a.† The EMF of the bare silver electrode was unstable and drifted after changing the KCl solution concentration. The EMF of the electrochemically prepared silver/silver chloride electrode showed a stable response to changes in chloride ion concentration. The EMF of the silver/silver chloride electrode prepared with a 1 mM PDTA·Fe(iii) solution for 2 min is shown in Fig. S2b.† The EMF became unstable during the course of the calibration experiments and started to drift after 40 min. After that, it showed similar behaviour to that of the bare silver electrode. One reason for the unstable EMF was the dissolution of silver chloride and exposure of bare silver at the surface because of insufficient silver chloride coverage. As the concentration of PDTA·Fe(iii) and reaction time increased, the EMFs of the silver/silver chloride electrodes prepared with the chemical deposition method became more stable, as shown in Fig. 2a and S2c, d.† Electrodes prepared with 2, 5, and 10 mM PDTA·Fe(iii) solutions for 2, 5, and 10 min showed EMFs similar to that of the conventional silver/silver chloride electrode prepared electrochemically, if the chloride ion concentration in solution was higher than 0.1 mM.

To characterize the EMF behaviours of the electrodes more quantitatively, the standard deviations, drifts, and hystereses of the EMFs of electrodes prepared under different conditions are shown in Fig. 2b–d. The standard deviations, drifts, and hystereses of the EMFs of electrodes prepared with 2, 5, and 10 mM PDTA·Fe(iii) solution were sufficiently small and comparable to those of the electrochemically prepared silver/silver chloride electrode. Those of the electrode prepared with 1 mM PDTA·Fe(iii) solution for 2, 5, and 10 min were large or showed a similar tendency to those of the bare silver electrode. The calibration curves for these electrodes are shown in Fig. S3a–e.† The correlation coefficients in the concentration range from 0.01 mM to 100 mM and the slope sensitivities of the calibration curves were calculated from these figures, and are shown in Fig. 2e and f, respectively.

The correlation coefficients and slope sensitivities of these electrodes were largely comparable to those of the electrochemically prepared silver/silver chloride electrode, except for the electrode prepared with 1 mM PDTA·Fe(iii) solution for 2 min. The slope sensitivities of these electrodes in the concentration range from 0.1 mM to 100 mM were around 46–52 mV per decade. The performance of the silver/silver chloride electrode prepared with 2 mM PDTA·Fe(iii) solution for 2 min was good enough to be used for the experiments in this study. On the other hand, when the concentration of a PDTA·Fe(iii) solution becomes higher and a reaction time becomes longer, the silver chloride layer becomes thicker, which would result in deterioration of accessibility of target DNA to the probe DNA and hybridization efficiency. The silver chloride layer should be as thin as possible to be compatible with the SAM, and a stable EMF should be maintained throughout the experiment and expected period of practical use as a disposable single-use sensor. The optimum conditions for preparing the silver chloride by chemical deposition were therefore determined to be 2 mM PDTA·Fe(iii) for 2 min.

### Characterization of the mixed SAM

In the present study, the mixed SAM was prepared on the surface of the silver film using oligonucleotide- and sulfobetaine (SB)-terminated alkanethiols. Details of the reaction scheme are given in the Materials and Methods section. To design a sensing surface with high signal to noise ratio, it is important to control the density of immobilized oligonucleotide probes. This maximizes the efficiency and kinetics of hybridization. Nonspecific adsorption by interfering molecules (non-complementary DNAs or interfering proteins) should also be minimized. In the previous studies, when 11-mercapto-1-undecanol (MCU) or 6-mercapto-1-hexanol (MCH) was backfilled into the pre-adsorbed oligonucleotide-terminated alkane thiolate to prepare mixed DNA/MCU or DNA/MCH SAM on a gold surface, the suppression of nonspecific adsorbed non-complementary DNA and improved capture kinetics of the target DNA were reported.^32–37^ One of the reasons for these results was considered to be the reorientation of oligonucleotide probes to a more upright configuration upon adding MCU,^36^ and the displacement of oligonucleotide-terminated alkane thiolate by MCU. These factors led to reduced steric and electrostatic hindrances arising from the tightly packed DNA monolayer.^37^ Similar effects for the mixed SAM can be expected to occur on the silver surface in the present study. SB-terminated alkanethiol was used to backfill the pre-adsorbed oligonucleotide-terminated alkanethiolate SAM, to further suppress nonspecific binding of interfering proteins on the SAM.

**Fig. 3:**
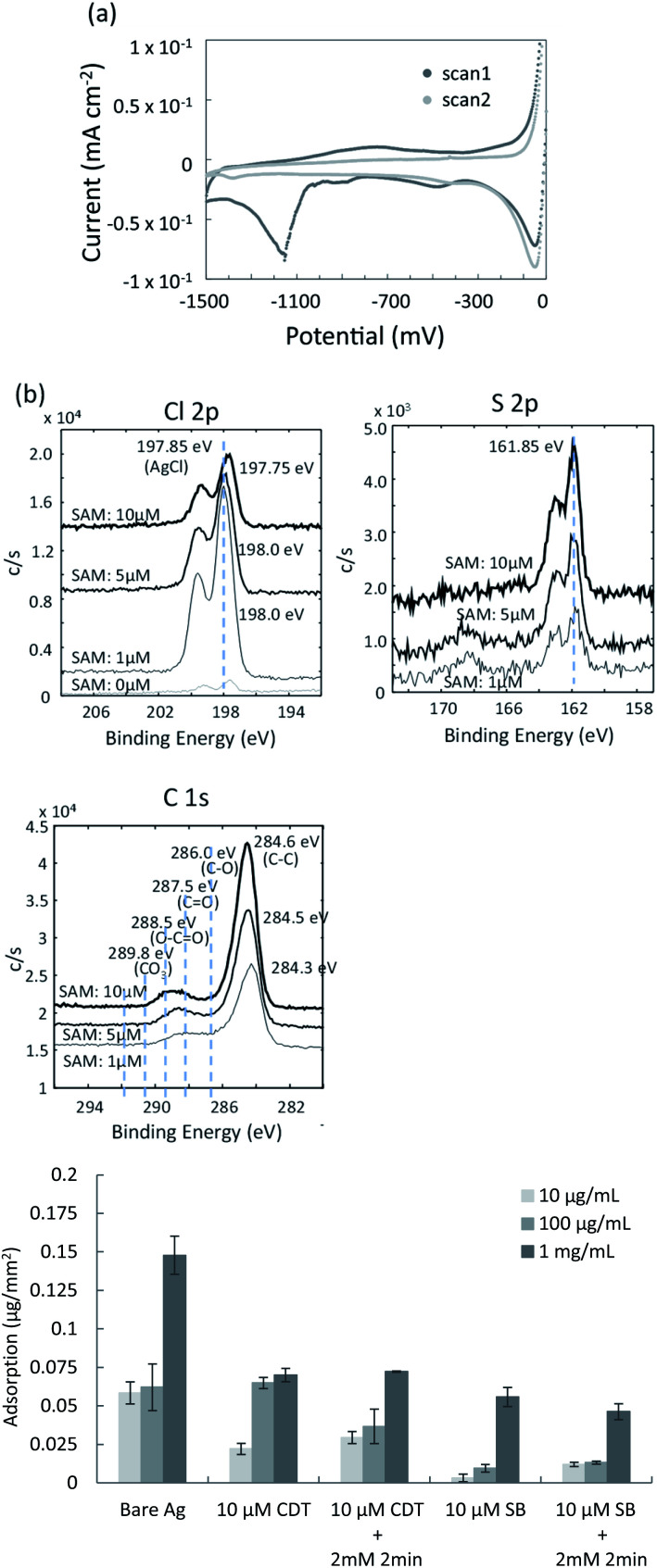
Characterization of co-deposition of mixed SAM and silver chloride on the silver thin film. (a) CV measurements of the mixed SAM on the sputtered silver thin film; (b) XPS spectra of mixed surfaces containing SAMs and silver chloride; (c) comparison of nonspecific protein adsorption for the various mixed surfaces.

The covalent bonding between thiol groups and the silver surface was confirmed by cyclic voltammetry (CV). The mixed SAM was prepared on the surface of the sputtered silver film using thiolated oligonucleotide probes and SB-undecanethiol. Typical CV curves for the SAM/Ag structure are shown in Fig. 3a. The reduction peak appeared around −1150 mV (*vs.* Ag/AgCl) for the SAM/Ag structure, while that for the SAM/Au structure appeared around −950 mV.^38^ This suggested that the affinity between sulphur and silver was stronger than that between sulphur and gold. The reduction peak at around −1150 mV in the first scan indicated the desorption of SB molecules from the silver surface. This peak was absent in the second scan because of the breaking of sulphur-silver bonds and irreversible desorption of SB. The SAM density was determined from the peak area (*Q*), which corresponded to the charge generated by the reduction of silver-thiol bonds based on eqn (5):^39^

**Fig. 4:**
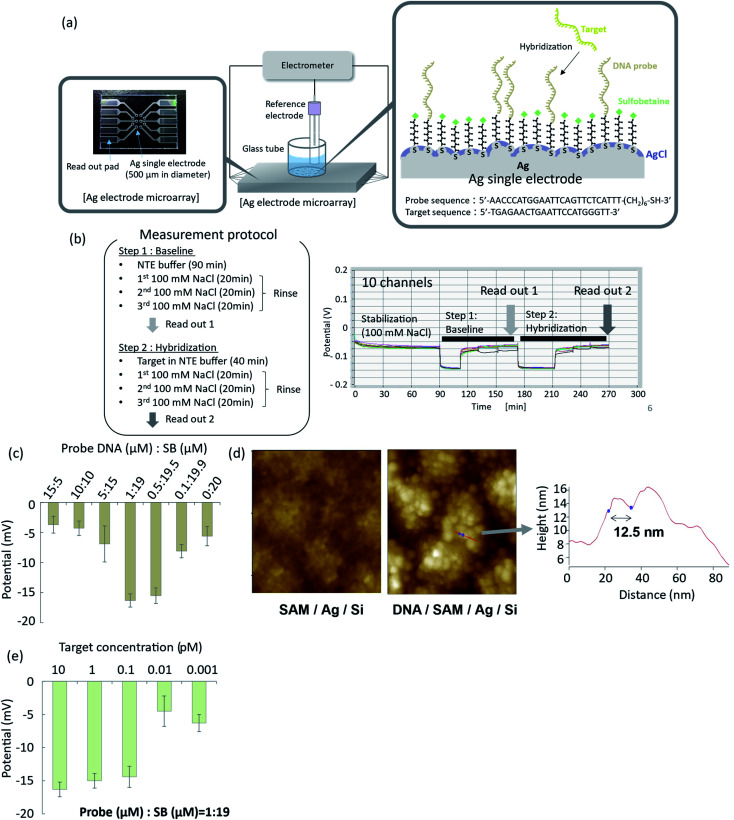
Potentiometric detection of DNA hybridization using organic and inorganic mixed phase modification of a silver surface. (a) Electrode array and experimental setup for potentiometric measurements; (b) time courses of EMFs of the electrode array during the hybridization procedure; (c) optimization of hybridization efficiency as a function of DNA probe : SB ratio; (d) AFM images of the modified surface with and without oligonucleotide probes; (e) effect of target DNA concentration on EMF response of the silver electrode with organic and inorganic mixed phase modification.

5 *γ* = *QN*_A_/2*FA*

where *Q*, *N*_A_, *F* and *A* are the peak area, Avogadro's number, Faraday's constant and the electrode surface area, respectively. According to the above relationship, the surface density of the SAM in Fig. 3a can be calculated to be 4.9 molecules per nm^2^. The S–S distance of the densely packed alkane thiolate on Ag(111) was reported to be approximately 0.46 nm,^5^ while that on Au(111) was approximately 0.5 nm,^5,40^ which corresponded to approximately 4 molecules per nm^2^. The thiolate monolayer on the Ag(111) substrate was more densely packed than the tilted thiolate monolayer on the Au(111) substrate because of the shorter S–S distance on Ag(111). Considering the surface roughness of the sputtered Ag thin film in the present study, the surface density of the mixed SAM shown in Fig. 4a was reasonable and indicated dense packing.

To confirm the co-deposition of the SAM and silver chloride layer on the surface of the silver thin film, elemental analyses were performed using X-ray photoelectron spectroscopy (XPS). The SAM of 10-carboxy-1-decanethiol (10-CDT) was formed on the sputtered silver thin film. This was followed by PDTA·Fe(iii) treatment for silver chloride deposition. 1 μM, 5 μM, and 10 μM solutions of 10-carboxy-1-decanethiol were used for SAM formation. High-resolution XPS spectra of the Cl 2p, S 2p, and C 1s regions are shown in Fig. 3b. The peaks for Cl 2p and S 2p were derived from AgCl and alkane thiolate, respectively. The C1 spectra indicated the C–C bond at 284.6 eV, C–O bond at 286.0 eV, C=O bond at 287.5 eV, and O–C=O bond at 288.5 eV. The peak intensity in the Cl 2p spectra decreased with increasing 10-CDT solution concentration, which indicated increasing surface coverage of the SAM. The peak intensities in the S 2p and C 1s spectra increased with increasing 10-CDT solution concentration because of the increasing SAM density. The XPS results were therefore consistent with the presence of the SAM and silver chloride layer on the surface of the silver thin film. The relative amounts of the SAM and silver chloride layer could be controlled by changing the concentration of the SAM solution.

### Evaluation of nonspecific adsorption

Zwitterionic SB has antifouling characteristics and can reportedly dramatically reduce nonspecific adsorption of proteins.^41,42^ To examine the antifouling effect of the co-deposition of the SAM and silver chloride layer, protein adsorption was evaluated using the organic and inorganic mixed phase modification with the 10-CDT SAM or sulfobetaine3-undecanethiol SAM. Fig. 3c shows the nonspecific adsorption of bovine serum albumin (BSA) on the different surfaces of the thin film silver substrates after direct contact with 10 μg mL^−1^, 100 μg mL^−1^, and 1 mg mL^−1^ BSA solutions. BSA adsorption on the surface of the SB SAM with and without chemical deposition of silver chloride was about one third to one forth compared with those on the surface of the 10-CDT SAM. Less BSA adsorption on the SB-terminated SAM was therefore confirmed, even after chemical deposition of silver chloride with 2 mM PDTA treatment for 2 min. This result indicated that the silver chloride layer was estimated to be thinner than the length of sulfobetaine3-undecanethiol, which was approximately 3 nm long.

### Potentiometric measurement of DNA hybridization

To demonstrate potentiometric measurements of hybridization, arrayed thin film silver electrodes were fabricated on glass substrates by radio frequency (rf) sputtering. Ten electrodes of 500 μm in diameter were integrated into a microarray, as shown in Fig. 4a. Details of the fabrication process are given in the Materials and Methods section. A mixed SB- and oligonucleotide-terminated SAM was formed on the silver surface, according to the method described in the previous section. A silver chloride layer was subsequently deposited by chemical deposition using 2 mM PDTA·Fe(iii) for 2 min of immersion. The EMFs of the functionalized electrodes were measured by an electrometer with a high-input impedance using an Ag/AgCl (in 100 mM KCl solution with a salt bridge) reference electrode. Typical time courses of the EMFs for the ten electrodes are shown in Fig. 4b. The ten electrodes were immersed in 100 mM NaCl solution and incubated for 1.5 h to achieve stabilization. A NaCl–tris–EDTA (NTE) buffer without the target oligonucleotide was then introduced, and the system was allowed to stand for 20 min (step 1), followed by rinsing with 100 mM NaCl solution. The EMFs of the ten electrodes were measured and averaged as a baseline signal. NTE buffer with different concentrations of target oligonucleotide was then introduced to the surface of the functionalized SAM, and the system was allowed to stand for 40 min to achieve hybridization (step 2). The hybridization buffer with the target oligonucleotide was then replaced by 100 mM NaCl solution for rinsing. The EMFs of the ten electrodes were then measured and averaged as the specific hybridization signal. The difference between the specific hybridization signal and base line signal was related to the hybridization event, so differential signals were evaluated in this study. When the oligonucleotide : SB ratio in the mixed SAM was 1 : 19, the standard deviation and drift of the EMFs for the ten electrodes in 100 mM NaCl solution were 0.25 mV and −1 × 10^−4^ mV min^−1^, respectively. These results confirmed that the EMFs had good uniformity and stability.

### Optimization of DNA probe density

To optimize hybridization efficiency, we performed hybridization experiments with changing mixed SAM ratio. Hybridization efficiency was expressed in terms of the EMF change after hybridization. As shown in Fig. 4c, high hybridization efficiency was obtained when the oligonucleotide : SB ratio at the surface was 1 : 19. The oligonucleotide probe density under this condition corresponded to 0.36 probes per nm^2^, as determined from CV measurements. This probe density is in good agreement with a reported value for which the maximum hybridization efficiency was obtained with the oligonucleotide/MCU SAM.^37^ The current probe density is also close to the maximum density for closely packed oligonucleotide probes on a rf-sputtered gold surface, which is reportedly 0.3–0.4 probes per nm.^2,43^ When the surface density of oligonucleotide probes is too high, oligonucleotide probes interfere with neighbouring probes *via* steric and electrostatic interactions, which degrades the hybridization efficiency. In some studies of mixed SAMs with oligonucleotide/MCH probes immobilized on gold surfaces, the surface density of oligonucleotide probes is reportedly 0.01–0.1 probes per nm.^2,32,44–47^

The AFM image of the prepared organic and inorganic mixed phase structure with oligonucleotide probes, observed in Tris–EDTA buffer, is shown in Fig. 4d. Globular structures with sizes of around 10 nm were observed on the surface of the silver chloride grains and in the narrow spaces between grains. These globular structures were not observed on the SAM surface without oligonucleotide probes. Single-stranded DNA probes were reportedly observed as globular structures in AFM analysis,^48–52^ so the globular structures in Fig. 4d were considered to be oligonucleotide probes. The length of the line indicated by the arrow in Fig. 4d was 12.5 nm, while the diameters of the globular structures were reported to be around 10 nm for single-stranded DNA with 25 bases,^48^ 8.4 nm for oligonucleotide with 20 bases,^49^ 5–10 nm for oligonucleotide with 33 bases,^51^ and 11 nm for single-stranded DNA with 12 bases.^52^ Although the length of the line in Fig. 4d was roughly in agreement with the reported diameters of single-stranded DNA probes, the diameters of the globular structures in Fig. 4d were not uniform. Instead, they were distributed in the range from several nanometres to several tens of nanometres. This suggested that a small number of oligonucleotide probes formed a domain or cluster at the surface of the silver thin film. The oligonucleotide probes immobilized in the narrow spaces between grains were not expected to effectively capture the target DNA because of insufficient space for efficient hybridization. Further investigation is necessary to clarify the detailed structure of the organic and inorganic mixed phase surface and kinetics of hybridization in these narrow spaces.

### Hybridization assay of microRNA

The relationship between the EMF change and target DNA concentration is shown in Fig. 4e. The standard deviations of the EMFs were sufficiently small, even after hybridization and washing with 100 mM NaCl solution. The limit of detection was determined based on the standard deviation of the EMF in the blank sample with the confidence value of 3. The 3SD value in the blank sample was −13.8 mV, while the average signal of the sensor in 0.1 pM of the target DNA was −14.4 mV, which was greater than the 3SD value. On the other hand, the average signal of the sensor in 0.01 pM of the target DNA was −4.5 mV, which was smaller than the 3SD value. Based on these results, the limit of detection was determined to be 0.1 pM for the organic and inorganic mixed phase structure. In Fig. 4e, the signal to noise ratio was 9.1 at a concentration of 0.1 pM, which was higher than that reported previously for potentiometric nucleic acid detection.^53^ These results demonstrated that stable and reliable detection of microRNA with high sensitivity was achieved using the organic and inorganic mixed phase surface.

When monitoring the sensors for an extended duration, the EMF sometimes became unstable when the Ag/AgCl layer was thin. For Ag/AgCl layers prepared with 2 mM PDTA·Fe(iii) for 2 min, the EMF was stable for the first 5 h, but afterwards sometimes became unstable. This may have resulted from the dissolution of silver chloride in the buffer solution containing 0.1 M NaCl, which leads to exposure of bare silver surface and degradation of the EMF stability over the course of the experiment. In most applications and especially for medical use, sensors for hybridization assays are usually single-use and disposable. For this purpose, the stability of the proposed sensor would be sufficient.

In the hybridization experiments, the complimentary DNA of microRNA 146a-5p (miR-146a) was employed as a model detection target. The miR-146a target carries important information in the progress of androgen-independent prostate cancer^54^ and has clinical significance in bladder cancer.^55^ The proposed detection method based on the organic and inorganic mixed phase modification of a silver surface would be useful for the simultaneous detection of several microRNAs and circulating tumour DNAs for liquid biopsies in clinical diagnoses.

## Conclusions

The conclusions section should come in this section at the end of the article, before the acknowledgements. Organic and inorganic mixed phase modification of a silver surface was used to achieve functionalization with biomolecules and stabilization of the EMF. A SAM of alkanethiol was formed on the surface of a sputtered Ag thin film, and the silver surface was then oxidized with PDTA·Fe(iii) to form silver chloride in the liquid phase. The resulting mixed structure was confirmed with XPS and CV. The composition of the organic phase and inorganic phase could be controlled by changing the concentration of the SAM solution. For the hybridization assay, a mixed SAM of oligonucleotide- and SB-terminated alkanethiols was formed together with silver chloride on the surface of the sputtered Ag thin film. The resulting modified surface reduced the nonspecific binding of interfering biomolecules and achieved a high signal to noise ratio. The lower detection limit of the target DNA was 0.1 pM, at which concentration the signal to noise ratio was 9.1. The proposed sensor with the organic and inorganic mixed structure could be operated with a stable EMF for at least 5 h. The stability sometimes deteriorated during extended operating duration, which was attributed to the dissolution of silver chloride. However, the sensor was sufficiently stable for use as a disposable single-use sensor in clinical diagnoses.

Quantitative information of nucleic acids such as microRNA and circulating tumour DNA in body fluids is a useful biomarker for the clinical diagnosis of cancer. Liquid biopsies based on such information are now frequently incorporated into clinical trials. There remains room for improvement in the reproducibility and reliability of chip-based assays in clinical settings. Solid-state potentiometric sensors are nonetheless well-suited for achieving simple, multiplexed, and miniaturized point-of-care testing.

## Materials and methods

### Materials

1,3-Diaminopropanetetraacetic acid ferric ammonium salt monohydrate (PDTA·Fe(iii)) was kindly donated by CHELEST Corporation and used as received. 10-Carboxy-1-decanethiol (10-CDT) and sulfobetaine3-undecanethiol (SB) were purchased from Dojindo Molecular Technologies, Inc. NaCl, KCl, HCl, albumin, from bovine serum, fraction V (BSA), and sodium dodecyl sulphate (SDS) were obtained from FUJIFILM Wako Pure Chemical Corporation. Tris-(2-carboxyethyl)phosphine (TCEP) was supplied by Sigma-Aldrich. Micro bicinchoninic acid (BCA) protein assay kit and Dulbecco's phosphate buffered saline (DPBS) were purchased from Life Technologies Co. Tris–EDTA (TE) buffer and NaCl–tris–EDTA (NTE) buffer were purchased from Promega and Nippon Gene Co., Ltd, respectively. Silver wire was purchased from Nilaco Corporation. Sputtered silver thin films on glass substrates and silver electrode arrays were fabricated using a NIMS (National Institute for Materials Science) fabrication platform. DNA oligonucleotide probes and DNA targets were provided by Tsukuba Oligo Service Co. Ltd.

### Electrochemical deposition of silver chloride

Silver wire (0.5 mm in diameter) was ultrasonically cleaned in acetone and ethanol for 10 min each, and then rinsed with ultra-pure water. The pre-treated silver wire was then etched in concentrated nitric acid for 1 min and rinsed with ultra-pure water immediately afterwards. The wire was then immersed in 0.1 M HCl solution, and the silver chloride layer was electrochemically deposited on the silver surface by applying 1 V against a platinum counter wire.

### Measurement of EMF for functionalized electrodes

The EMFs of the bare silver wire, electrochemically deposited silver chloride wire, and chemically deposited silver chloride wire were measured in KCl solutions with concentrations ranging from 0.01 mM to 100 mM. In brief, the potentiometric responses of the electrodes as a function of KCl concentration were evaluated using an Ag/AgCl (in 100 mM KCl solution with a salt bridge) reference electrode with a high-input impedance electrometer (Keithley 6517B, Cleveland, OH, USA) at no DC bias voltage. The EMFs of the electrodes were recorded during increasing and decreasing KCl concentrations (0.1 mM–100 mM–0.1 mM) for 10 min each at 25 °C (Fig. 2a). The average EMF and the standard deviation at each KCl concentration were calculated from five sets of measurement data.

### XPS analyses

For high-resolution XPS analysis, a 90 nm-thick silver layer was deposited with Jsputter (ULVAC, Inc., Kanagawa, Japan) on a glass substrate after surface treatment by reverse sputtering and deposition of a 10 nm-thick Ti layer. For preparing SAMs on the sputtered silver thin film, 1, 5, and 10 μM of 10-CDT ethanol solution was deposited dropwise on the surface, and the substrates were kept at room temperature under dark conditions for about 15 h. Subsequently, the 10-CDT SAM immobilized silver surface was treated with PDTA·Fe(iii) dissolved in 100 mM NaCl solution for chemical deposition of the silver chloride layer, without applying a voltage. Surface elemental analysis by XPS was performed using a PHI Quantera SXM instrument (ULVAC-PHI, Inc., Kanagawa, Japan) equipped with a 20 kV Al-Kα radiation source at the anode.

### Protein adsorption experiments

Nonspecific adsorption of protein on the SAM surface was evaluated using the micro BCA method according to the manufacturer's protocol. Silver wire was immersed in 10 μM 10-CDT ethanol solution or 10 μM SB aqueous solution to form the SAM, and then treated with 2 mM PDTA·Fe(iii) for 2 min to deposit silver chloride. To prepare sample solutions for the micro BCA method, the SAM immobilized wires were immersed in a tube containing 10 ng mL^−1^, 1 μg mL^−1^, or 100 μg mL^−1^ of BSA solution for 30 min, and then ultrasonically washed in 1 wt% SDS solution for 3 min to remove adsorbed BSA. The collected sample solutions were mixed with the BCA working reagents and incubated at 37 °C for 2 h. Subsequently, the absorbance was measured at 562 nm with a plate reader (Infinite 200 PRO, Tecan Japan Co., Ltd., Kanagawa, Japan). The standard curve was prepared in advance by plotting the absorbance intensities of BSA standard solutions at 562 nm in the concentration range from 0 (blank) to 40 μg mL^−1^. The BSA concentration of each sample solution was then determined from the standard curve.

### Organic and inorganic mixed phase modification of the silver surface

A glass tube with an inner diameter of 6.8 mm and length of 10 mm was attached over the thin film silver electrodes of a 10-channel microarray, as shown in Fig. 4a, using a thermosetting resin (ZC-203, Nippon Pelnox, Tokyo, Japan). The mixed SAM was formed on each silver electrode of the microarray by the two-step chemisorption of complementary DNA specific to hsa-miR-146a-5p and SB. Briefly, oligonucleotide-terminated alkanethiols (SH-C6-oligoDNA probe (5′-AACCCATGGAATTCAGTTCTCATTT-(CH2)6-SH-3′)) of various concentrations dissolved in TE buffer were adsorbed on the silver electrodes. The remaining silver surfaces were backfilled with SB-terminated alkanethiolate by immersing in SB aqueous solution containing TCEP at the same molar concentration as SB for 15 h, followed by rinsing with pure water. Hereby, the molar ratio (μM) of the DNA probe to SB in the mixed SAM was varied in the following manner, 20 : 0, 15 : 5, 10 : 10, 5 : 15, 1 : 19, 0.5 : 19.5, 0.1 : 19 : 9, 0 : 20, so that the total concentration was equal to 20 μM. The SAM density was confirmed using CV with a three-electrode system of a mixed SAM immobilized silver working electrode, platinum counter electrode, and Ag/AgCl reference electrode (in 100 mM KCl with a salt bridge). Degassed 0.1 M KOH aqueous solution was added into a glass chamber, and the Pt counter electrode and Ag/AgCl reference electrode were introduced to the chamber. The voltage scan was performed over a potential window from 0 V to −1.5 V, and back to 0 V at a scan rate of 0.1 V s^−1^ at room temperature using an Autolab PGSTAT 302 apparatus (Eco Chemie, Utrecht, Netherlands). The SAM density was determined using the area of the reduction peak at around −1150 mV in the first scan of the voltammogram with a linear baseline correction using the bundled software. After immobilization of the mixed SAM, chemical deposition of the silver chloride layer was performed in 2 mM PDTA·Fe(iii) solution for 2 min.

### Hybridization assays

Hybridization between the DNA probe and target (5′-TGAGAACTGAATTCCATGGGTT-3′) was confirmed from the EMF responses of the functionalized electrodes. The electrodes of the microarray were connected to a high-input impedance electrometer (6517B, Keithley Instruments, Cleveland, OH, USA) with a low current, 10-channel scanner card (Model 6522, Keithley). Ag/AgCl (in 100 mM KCl solution) was used as a reference electrode *via* a salt bridge. The electrodes were incubated in 100 mM NaCl solution for 90 min at 25 °C. The solution was changed to NTE buffer (no target) and replaced three times by 100 mM NaCl solution. The baseline signal was obtained as the average EMF of the 10 electrodes using 5 sampling points before the hybridization step, as shown in Fig. 4b. The solution was then changed to the NTE buffer with 20 pM target DNA, incubated for 40 min for hybridization, and replaced three times by 100 mM NaCl solution. The specific hybridization signal was obtained as the average EMF of the 10 electrodes using the last 5 sampling points during the rinsing process. The difference between the specific hybridization signal and base line signal was evaluated as an index for the hybridization efficiency.

## Author contributions

Y. M. conceived and coordinated the research. M. T. and Y. M. designed and performed most of the experiments and analyzed the data. Y. M. M. T. and K. N. performed the electrochemical measurements of the devices. D. T. fabricated the silver electrode array device. M. T., C. K.-H., T. G., A. M. and Y. M. contributed to the interpretation of the data. Y. M. and M. T. wrote the manuscript. All authors have given approval to the final version of the manuscript.

## Conflicts of interest

There are no conflicts to declare.

## Supplementary Material

RA-011-D1RA03449A-s001
