# Supplementary material for: Organic and inorganic mixed phase modification of a silver surface for functionalization with biomolecules and stabilization of electromotive force
Source: RSC Adv. 2021 Jul 16;11(40):24958–67. doi: 10.1039/d1ra03449a (PMC9036890; doi:10.1039/d1ra03449a)
Supplement: RA-011-D1RA03449A-s001 [file RA-011-D1RA03449A-s001.pdf]

Electronic Supplementary Material (ESI) for RSC Advances.
This journal is © The Royal Society of Chemistry 2021

Supplementary Information

Organic and inorganic mixed phase modification of silver surface for functionalization with biomolecules and stabilization of electromotive force

Miyuki Tabata,[a] Chiho Kataoka-Hamai, [b] Kozue Nogami, [a] Daiju Tsuya, [b] Tatsuro Goda,[a†] Akira Matsumoto [a] and Yuji Miyahara [a]

[a] Tokyo Medical and Dental University, 2-3-10 Kanda-Surugadai, Chiyoda, Tokyo 101-0062 Japan

[b] National Institute for Materials Science, 1-2-1 Sengen, Tsukuba, Ibaraki, 305-0047 Japan

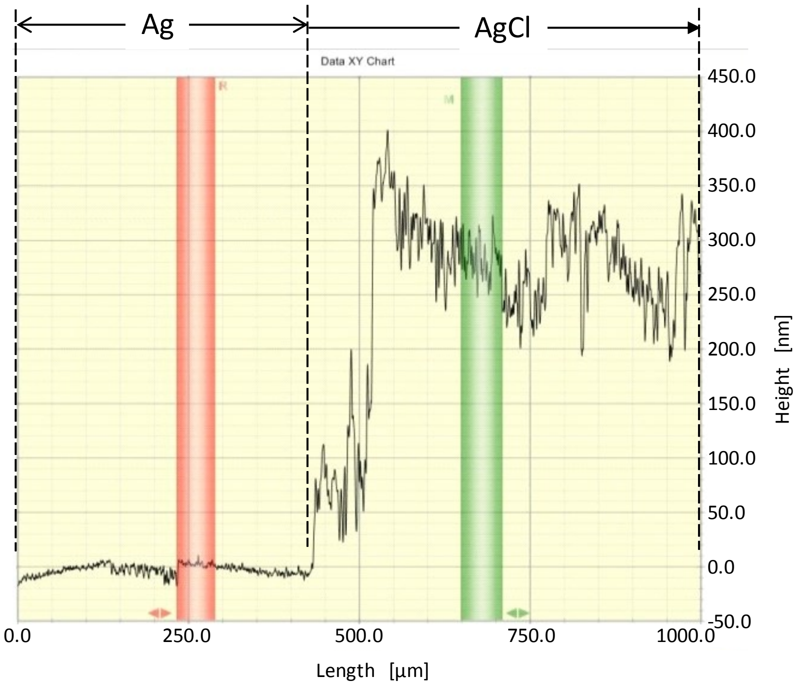

Figure S1. Thickness measurement of silver chloride layer prepared with 200 mM PDTA・Fe(III) for 180 sec.

Part of the sputtered silver thin film was covered with Kapton tape, and silver chloride was formed at the surface of the silver layer. The Kapton tape was then removed, and the step at the boundary between the silver surface and silver chloride surface was scanned with the needle tip. Fig. S1 shows the scan result of the silver chloride layer prepared with 200 mM PDTA・Fe(III) for 180 sec. The data between 233.3 µm and 290.7 µm (red area), and between 649.9 µm and 711.7 µm (green area) were averaged, respectively, and the difference between these two averages was regarded as the thickness of the silver chloride layer. From Fig. S1, the thickness of the silver chloride layer was found to be 278.4 nm.

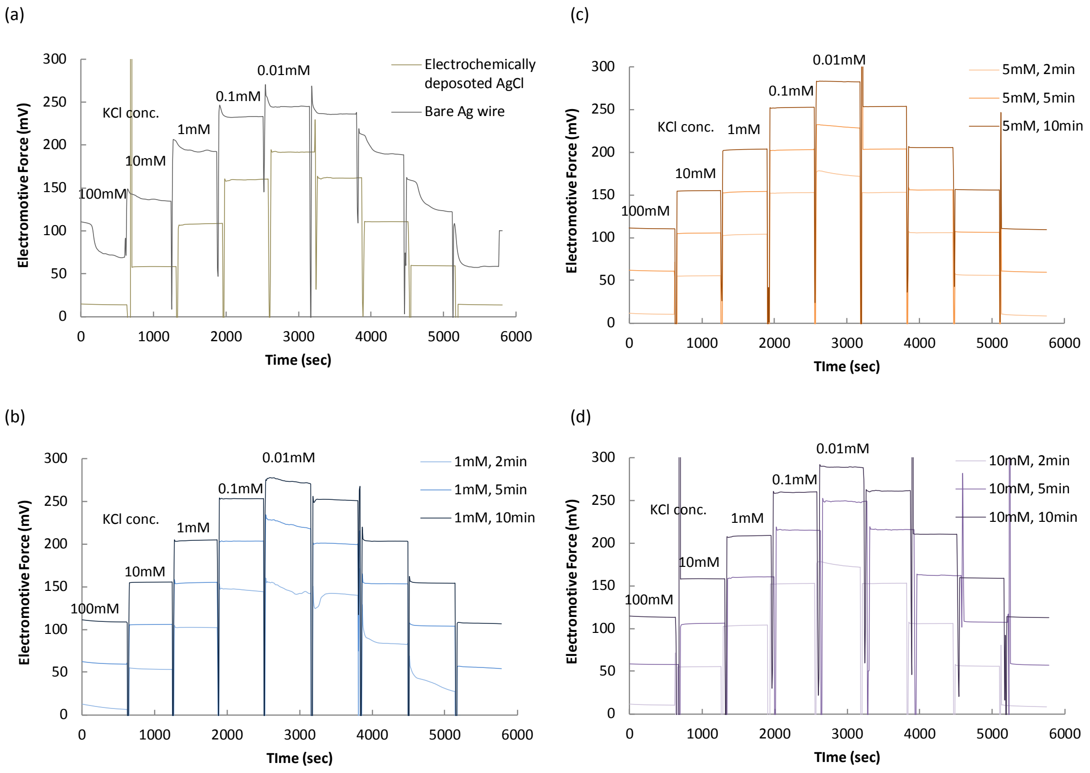

Figure S2. EMFs of silver/silver chloride electrodes prepared under different conditions.

Typical raw data for the EMFs of silver/silver chloride electrodes in response to various concentrations of potassium chloride in the range from 0.01 mM to 100 mM are shown in Fig. S2. The response curve for each electrode is shown offset on the voltage axis for clarity. The silver/silver chloride electrodes were prepared with 1, 5, and 10 mM PDTA・Fe(III) solution for 2, 5, and 10 min. The EMF behaviours of a bare silver electrode and an electrochemically deposited silver/silver chloride electrode are shown in Fig. S2a for comparison.

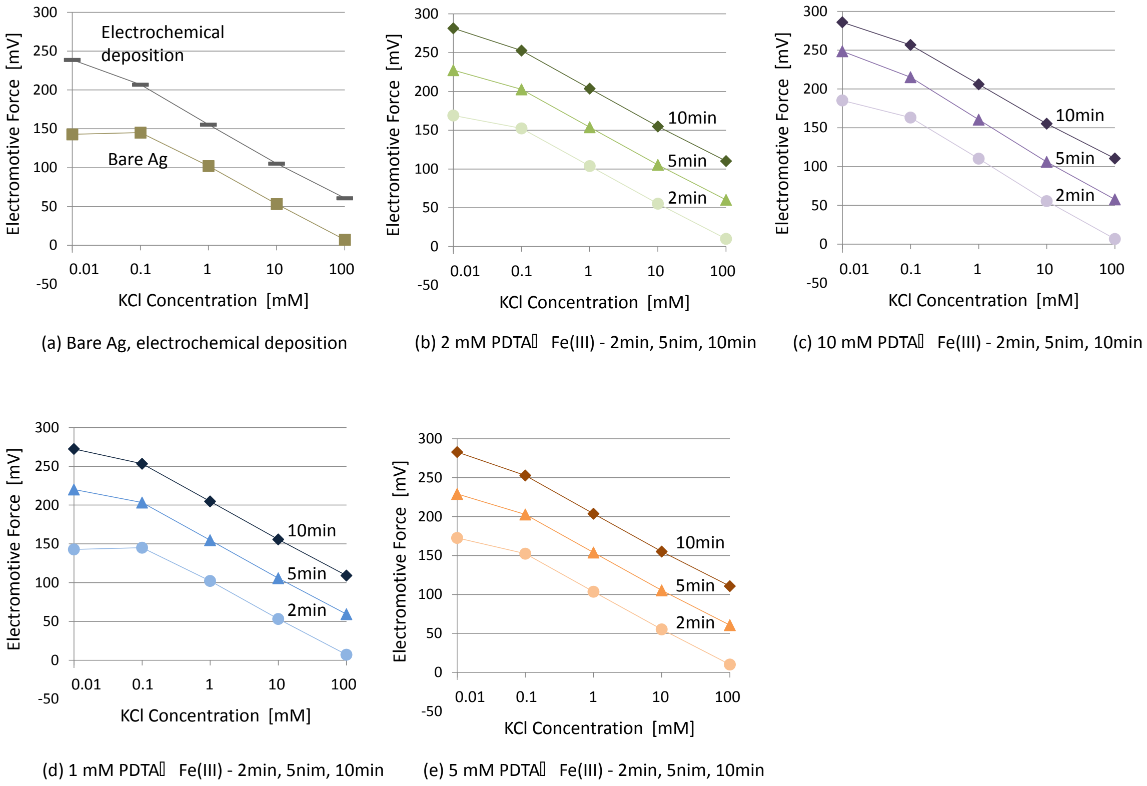

Figure S3. Calibration curves for chemically prepared silver chloride electrodes.

Calibration curves for silver/silver chloride electrodes prepared with 1, 5, and 10 mM PDTA・Fe(III) solutions for 2, 5, and 10 min, are shown in Fig. S3a–e. From these figures, the correlation coefficients in the concentration range from 0.01 mM to 100 mM and the slope sensitivities of the calibration curves were calculated, and are shown in Fig. 2e and f, respectively.

Ideally, the EMF responses of the Ag/AgCl electrodes should follow the Nernst equation expressed by equation (S1),

$$E = E_0 + \left(2.303\,{}^{RT}\!/_{zF}\right)\log a \qquad \text{(S1)}$$

where E_0 is the standard electrode potential, R the ideal gas constant, T the temperature in Kelvin, z the number of moles of electrons, F is the Faraday constant, and a is the ion activity. At 25 °C, the EMF change in response to a decade change in chloride ion concentration is expected to be –59.2 mV/decade.
